# The Emerging Role of CT-Based Imaging in Adipose Tissue and Coronary Inflammation

**DOI:** 10.3390/cells10051196

**Published:** 2021-05-13

**Authors:** Jeremy Yuvaraj, Kevin Cheng, Andrew Lin, Peter J. Psaltis, Stephen J. Nicholls, Dennis T. L. Wong

**Affiliations:** 1 Monash Cardiovascular Research Centre, Victorian Heart Institute, Faculty of Medicine, Nursing and Health Sciences, Monash University and Monash Heart, Monash Health, Clayton, VIC 3168, Australia; jeremy.yuvaraj@monash.edu (J.Y.); kevin.cheng@monash.edu (K.C.); stephen.nicholls@monashhealth.org (S.J.N.); 2 Cedars-Sinai Medical Center, Biomedical Imaging Research Institute, Los Angeles, CA 90048, USA; andrewklin@gmail.com; 3 Department of Medicine, University of Adelaide, Adelaide, SA 5005, Australia; peter.psaltis@sahmri.com; 4 South Australian Health Medical Research Institute, Adelaide, SA 5000, Australia

**Keywords:** atherosclerosis, coronary artery disease, computed tomography coronary angiography, coronary inflammation, adipose tissue, epicardial adipose tissue, pericoronary adipose tissue

## Abstract

A large body of evidence arising from recent randomized clinical trials demonstrate the association of vascular inflammatory mediators with coronary artery disease (CAD). Vascular inflammation localized in the coronary arteries leads to an increased risk of CAD-related events, and produces unique biological alterations to local cardiac adipose tissue depots. Coronary computed tomography angiography (CTA) provides a means of mapping inflammatory changes to both epicardial adipose tissue (EAT) and pericoronary adipose tissue (PCAT) as independent markers of coronary risk. Radiodensity or attenuation of PCAT on coronary CTA, notably, provides indirect quantification of coronary inflammation and is emerging as a promising non-invasive imaging implement. An increasing number of observational studies have shown robust associations between PCAT attenuation and major coronary events, including acute coronary syndrome, and ‘vulnerable’ atherosclerotic plaque phenotypes that are associated with an increased risk of the said events. This review outlines the biological characteristics of both EAT and PCAT and provides an overview of the current literature on PCAT attenuation as a surrogate marker of coronary inflammation.

## 1. Introduction

In recent decades, atherosclerosis has become well recognised as a disease of chronic vascular inflammation. Where the excess accumulation of lipid within layers of the arterial wall was thought to be the most prominent driver of plaque formation, a strong body of evidence, from histological studies to clinical trials, highlights the critical effect of vascular inflammatory mechanisms in plaque formation and morphology, as well as their contribution to the onset of major coronary events. The poor localisation of traditional systemic biomarkers to the coronary vasculature has led to exploration and discovery of suitable alternative methods of quantifying inflammatory risk using non-invasive coronary computed tomography angiography (CTA) imaging.

## 2. Inflammation in Atherosclerosis

The nature of inflammation within atherosclerosis consists of a complex interplay of cells and mediators derived from both innate and adaptive immunity [1,2]. Pathological mechanisms driving plaque formation involve the accumulation of low-density lipoprotein (LDL) within the subendothelial space, which undergoes modifications to enhance immunogenicity. Under conditions of LDL excess, however, resident macrophages and smooth muscle cells become engorged with lipid, and undergo transformation into highly necrotic foam cells, which manifest macroscopically as the precursors to plaque in ‘fatty streaks’. This triggers the induction of a pro-inflammatory microenvironment via the upregulation of mediators facilitating further leukocyte localisation, chemotaxis and activation. For this reason, macrophages are considered the primary effectors of inflammation in atherosclerosis [1,3]. Chemokines and cell adhesion molecules stimulate migration of monocytes to the site of injury, and upon arrival differentiate into pro-inflammatory M1 macrophages via the action of local secreted mediators such as monocyte-colony stimulating factor (M-CSF) and interferon-gamma (IFN-γ) [4]. IFN-γ is primarily secreted by pro-inflammatory T-helper 1 (Th1) cells, which form the major adaptive component to immune activity in atherosclerosis. Naïve T-cells are recruited and differentiate primarily into the Th1 phenotype under the effects of antigen-presenting cells, triggering IFN-γ generation whilst simultaneously inhibiting differentiation alternative T-cell lineages and ensuring T-cell retention at the lesion site. In addition, atherosclerotic plaque is linked with the presence of other lymphocytes, such as CD8^+^ T-cells, B-cells and natural killer cells, collectively evidencing the contribution of both innate and adaptive immunity to plaque inflammation [5,6,7]. Ultimately, a focal inflammatory microenvironment, induced by the persistence of both leukocytes and LDL in atherosclerotic plaque, propagates this localised inflammatory response to chronicity.

In addition to these experimental and observational studies that shed light on the biological subtleties of atherosclerotic inflammation, randomised clinical trials (RCTs) have established the association of inflammation with major cardiac events. The REVERSAL and PROVE-IT trials saw high-intensity statin intervention mediating a significant reduction in C-reactive protein (CRP) as well as endpoints in plaque progression and the onset of acute coronary syndrome (ACS) [8,9]. The CANTOS and COLCOT trials subsequently demonstrated that anti-inflammatory agents facilitated significant reductions in cardiac event risk [10,11,12]. Thus, increased vascular inflammation burgeons the aetiology of atherosclerosis from plaque formation to major coronary events.

## 3. Biology of Human Adipose Tissue

Human adipose tissue is a highly active endocrine organ with roles in energy storage and metabolism [13]. The presence of both adipocytes and stromovascular cells enables adipose tissue to act as a reserve of adipokines readily secreted into neighbouring tissues and vessels. Adipocytes, in particular, constitute the major fraction of cells within adipose tissue and play a critical role in maintaining healthy metabolic operation.

### 3.1. Adipose Tissue Depots

In humans, adipocytes are stratified into subtypes differentiable by their visual colouration to the naked eye and their function. White adipocytes comprise white adipose tissue (WAT), the most abundant adipose tissue subtype in humans, serving the chief role of energy storage. WAT derives circulating glucose and free fatty acids (FFAs) as substrates for lipogenesis, while also readily mobilising stored energy as FFAs that are released into the vasculature [13]. In contrast, brown adipose tissue (BAT) is responsible for energy expenditure and thermogenesis. Brown adipocytes are characterised by the abundance of mitochondria expressing uncoupling protein-1 (UCP1), enabling oxidation and clearance of circulating FFAs and, therefore, BAT plays an integral role in regulating metabolic function [14]. Additionally, the presence of UCP1-expressing adipocytes within WAT depots has led to the recognition of ‘brown-in-white’ or beige adipose tissue as a unique bevy of adipocytes distinct from both brown and white fat. Beige adipocytes are most prominently characterised by their plasticity, phenotypically resembling WAT in their latent state but upon activation adopting energy-expending functionality similar to their counterparts in BAT [15]. Human adipose tissue, therefore, demonstrates significant heterogeneity in morphological characteristics and roles performed by its constituent subpopulations in metabolic homeostasis.

These adipose tissue subtypes vary in localisation according to depots in the human body. Subcutaneous adipose tissue (SCAT) is a WAT-dominant depot that constitutes approximately 80% of total adipose tissue volume in the body, and acts as a physical buffer against mechanical stress, infection and heat loss, as well as being a natural reservoir for excess energy in the body [16]. Visceral adipose tissue (VAT), conversely, lies within the thoracic and peritoneal cavities and comprises a concomitantly smaller proportion of total fat volume [17]. VAT characteristically shares arterial supply and venous drainage with neighbouring anatomical structures, such that it bears an indirect association with the viscera through shared components of the vasculature. In the abdomen, for instance, VAT empties into the portal circulation alongside other abdominal organs, and through the portal circulation is able to modulate hepatic metabolism [18]. Thus VAT not only shares immediate proximity to major viscera and vessels, but also possesses a critically important capacity to regulate metabolic operation in these regions in a paracrine manner.

### 3.2. Visceral Adipose Tissue of the Heart

While multiple VAT sites may be found in the thorax, understanding the relationship between these depots and the vasculature requires distinguishing them from one another according to their position relative to the heart. Epicardial adipose tissue (EAT) is an anatomically and functionally distinct cardiac fat depot, situated entirely within the pericardial sac between the visceral pericardium and the epicardial surface of the heart [19]. As a VAT depot that directly borders the coronary vasculature without fascial border, it is important to understand the unique composition and functionality fulfilled by EAT under both healthy and diseased conditions.

**Figure 1:**
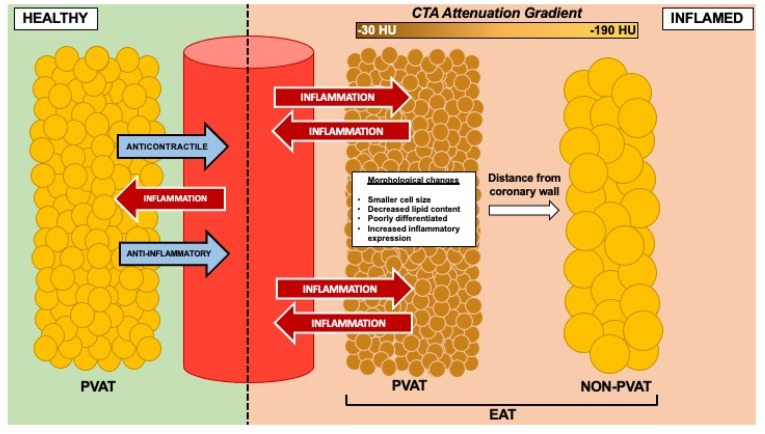
Schematic representation of bidirectional signalling between the vasculature and adipose tissue, particularly perivascular (or pericoronary) fractions of epicardial adipose tissue (EAT). Under ‘healthy’ conditions, perivascular adipose tissue (PVAT) typically exhibits anticontractile and anti-inflammatory properties in response to markers of oxidative stress and inflammation from the vasculature. Under ‘inflamed’ conditions, PVAT undergoes unique phenotypic changes due to vascular inflammation compared to non-perivascular adipose tissue located further from the vessel wall. These changes are detectable as increased attenuation on coronary computed tomography angiography (CTA).

On a cellular level, EAT is largely composed of smaller and more numerous adipocytes than abdominal VAT, even in the context of obesity [16,20,21]. Epicardial adipocytes exhibit characteristics of beige adipocytes, having greater rates of both fatty acid synthesis and digestion [21,22]. FFA oxidation accounts for a vital source of energy in the myocardium [23], and as an active source of FFAs EAT therefore plays a vital role in regulating cardiometabolic function. The proximity of EAT to the coronary vasculature and myocardium provides multiple means by which fatty acid migration can occur, in a mechanism similar to the previously discussed “portal theory” involving intraperitoneal VAT [18,19]. Of particular interest, therefore, are fractions of EAT that are ‘perivascular’ in nature, or immediately external to the vascular wall. Compositionally, perivascular adipose tissue (PVAT) depots in the body reflect the morphological peculiarities of neighbouring adipose tissue sites. For example, thoracic PVAT is thought to phenotypically resemble BAT, unlike abdominal PVAT which may harbour more WAT characteristics [24]. Perivascular components of EAT may therefore be constituted of primarily beige adipocytes, able to fulfil dynamic roles in FFA sequestration and elimination alongside the release of various vasoactive substances. The precise composition of adipocytes in this specific subset of EAT, however, remains unclear. Functionally, PVAT around the body modulates vascular tone by exerting anticontractile effects on the vasculature, but this is attenuated by exposure to local inflammation [25,26]. Importantly, these findings highlight the paracrine molecular interactions occurring between fat and vessel, as well as measurable changes occurring within adipose tissue consequent of inflammation (Figure 1).

## 4. Association of Inflammation and Cardiac Adipose Tissue

The signalling relationship shared between adipose tissue and vasculature is one that warrants significant consideration to understand the role of fat depots in metabolic regulation. The nominal function of human adipose tissue becomes deranged under adverse cardiometabolic conditions such as obesity, where increased triglyceride storage manifests in enlarged and hyperplastic white adipocytes associated with an amplified pro-inflammatory profile [27]. This particular interaction between adipose tissue and vasculature, therefore defines an ‘outside-inside’ signalling hypothesis, in which adipokines and other fat-derived mediators elicit changes within the vasculature. However, this relationship is not simply unidirectional, as emerging evidence suggests that mediators derived from the vessel wall effects distinct changes to adipose tissue on a local basis. In addition to observational PVAT studies discussed previously, Margaritis et al. [28] established that adiponectin is the crux of a unique crosstalk occurring between vasculature and local adipose tissue. The authors showed that circulating vascular superoxide production was predictably associated with lower levels of circulating adiponectin, as absence of the latter leads to uncoupling and dysfunction of endothelial nitric oxide synthase (eNOS). However, oxidative stress, represented by 4-hydroxynonenal (4-HNE), induced elevated rather than decreased adiponectin gene expression in local PVAT. Where 4-HNE has been previously described to decrease adiponectin expression as well as promote ubiquitin-mediated degradation of adiponectin in mice [29], it is clear that the opposite effect is observed in PVAT, leading the authors to surmise that production of adiponectin by local perivascular adipocytes is regulated by factors independent of that of other adipose tissue depots. This local interaction therefore highlights that it is not only ‘outside-inside’ signalling in the form of dysfunctional adipocytes promoting vascular inflammation that is present between fat and vessel, but ‘inside-outside’ signalling through local PVAT changes in response to vascular inflammatory markers (Figure 1).

### 4.1. Local Epicardial Adipose Tissue (EAT) Inflammation in Coronary Artery Disease (CAD)

This notion is one that may be further explored in the context of coronary artery disease (CAD). EAT exhibits a significantly higher inflammatory profile in patients with CAD, which may be exacerbated by acute inflammatory states such as cardiac surgery or myocardial infarction (MI) [30,31,32]. A seminal study by Antonopoulos et al. [33] found that perivascular adipocyte differentiation genes varied in expression depending on distance from the vasculature and exposure to inflammatory cytokines, emphasising the notion of differential adipose tissue properties relative to inflammation and proximity to the vessel wall. Adipocytes found closer to coronary adventitia had decreased expression of differentiation markers, manifesting in significantly smaller cell size and greater cell numbers, as well as a decreased lipid content both in vivo and ex vivo. The authors found that these phenotypic alterations were quantifiable using coronary CTA attenuation of adipose tissue around the coronary arteries, in which increased coronary CTA attenuation corresponds to morphological PVAT changes associated with coronary inflammation, while ‘healthy’ adipocytes bereft of exposure to inflammation are distinctly less attenuated. Moreover, inflammatory changes are most profound in perivascular adipocytes, with an attenuation gradient being mapped between adipocytes proximal and distal to the adventitia [33].

### 4.2. Adipose Tissue Inflammation on Positron-Emission Tomography-Computed Tomography (PET-CT) Imaging

Non-invasive imaging is capable of visualising the unique CAD-induced inflammatory changes occurring within adipose tissue. Evidence for focal inflammation within adipose tissue has arisen from studies of hybrid positron-emission tomography-computed tomography (PET-CT). PET-CT maps local uptake of radioactive tracers within specific sites of interest, such as adipose tissue [34], therein providing real-time assessment of metabolic and inflammatory activity. 18F-Fluorodeoxyglucose (18F-FDG) is a well-validated exemplar that reflects glucose uptake in regions of high inflammatory activity. In CAD, 18F-FDG on PET-CT has demonstrated increased adventitial and PVAT inflammation in coronary segments with plaque [35,36,37]. Limitations associated with myocardial ‘spill-over’ uptake of 18F-FDG hinders interpretability in a significant proportion of patients, leading to exploration of other tracers demonstrating usefulness in inflammatory assessment, such as sodium fluoride [38,39]. The clinical utility of PET-CT is hindered most significantly by its cost and limited distribution. It has, nevertheless, highlighted the activity of inflammation in adipose tissue and its relationship to coronary plaque, and that inflammatory adipose tissue changes can be envisaged using specific non-invasive imaging techniques. Coronary CTA attenuation has been shown to agree with PET-CT in detecting inflamed adipose tissue [33,35] and ‘vulnerable’ coronary plaques [37,40], and has formed the basis of growing investigation into the utility of assessing PVAT-specific changes as a surrogate marker of coronary inflammation.

## 5. Coronary Computed Tomography Angiography (CTA) Assessment of EAT and Pericoronary Adipose Tissue (PCAT) as a Marker of Coronary Inflammation

Coronary CTA has expanded in its use and is now among the most affordable and widely-distributed means of non-invasive imaging, with validated applications in the context of CAD. Technological advancements, such as iterative reconstruction algorithms and increased number of detector rows, have afforded coronary CTA with a variety of improvements that enhance its utility in a clinical setting, providing higher quality acquisitions with lower image noise and radiation dose [41]. In contrast to the real-time assessment provided by PET imaging, coronary CTA provides primarily anatomical information on architectural changes to the myocardium and coronary vasculature, the latter of which includes those associated with atherosclerotic plaque. It may be partially disadvantaged by an inherently inferior spatial resolution compared to invasive imaging techniques, as well as the presence of imaging artefacts arising from high-radiodensity structures, such as calcified plaque or coronary stents [42,43]. Nevertheless, coronary CTA provides a wide variety of structural information to the reader, including that pertaining to adipose tissue characteristics. Not only is coronary CTA highly accessible at relatively low clinical cost, routine CT images are able to be investigated retrospectively, providing numerous avenues through which inflammatory changes in the context of CAD may be explored.

### 5.1. EAT Quantification

**Figure 2:**
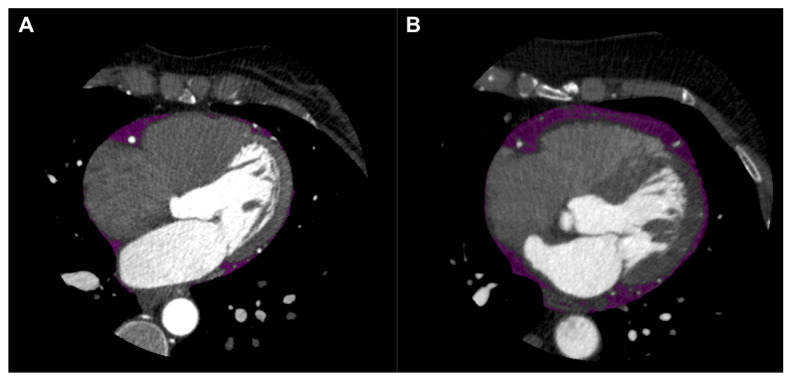
Epicardial adipose tissue (EAT) shown in purple on axial view of coronary computed tomography angiography (CTA). EAT in patient without coronary artery disease (CAD) shown in left panel (**A**), and EAT in patient with CAD shown in right panel (**B**).

CT attenuation of adipose tissue reflects morphological derangements experienced by adipocytes exposed to the effects of local vascular inflammation. Coronary CTA readily quantifies EAT volume and density as independent markers of adverse cardiometabolic risk each bearing associations with CAD (Figure 2) [44,45]. Increased EAT volume is a predictor of the presence of CAD, acute MI and ‘high-risk’ CAD phenotypes [45,46,47]. EAT density or attenuation has likewise been associated with CAD in numerous observational studies, but the broad nature of this marker in CAD remains highly heterogeneous thus far. With increasing coronary artery calcification (CAC), for example, EAT attenuation has been shown to either decrease [48,49,50] or increase [51,52]. Moreover, one study reported increased EAT attenuation predicted AMI [44], while another found decreased EAT attenuation predicted coronary events [48]. Statin therapy has been shown to decrease EAT attenuation without changes to serum lipid in a cohort of patients with limited CAC [53], while a prospective study evaluating patients with subclinical CAD found no impact of statins on EAT attenuation [54]. Such variance in the reported results of these studies may be owed to the nature of EAT as a depot, which inherently encompasses a wide range of adipocytes of varying proximity to the vessel wall and, accordingly, exposure to coronary inflammation. It is plausible, therefore, that inflammatory changes occurring in patients with CAD are mapped to a more limited degree in remote regions of EAT relative to perivascular adipocytes. Support for this notion resides in the fact that in one study [50], decreased EAT attenuation was most strongly associated with coronary calcification in a model adjusted for coronary artery bypass graft (CABG) surgery, suggesting differential effects of coronary calcification and acute inflammatory states on adipose tissue radiodensity.

### 5.2. Pericoronary Fat Attenuation Index

**Figure 3:**
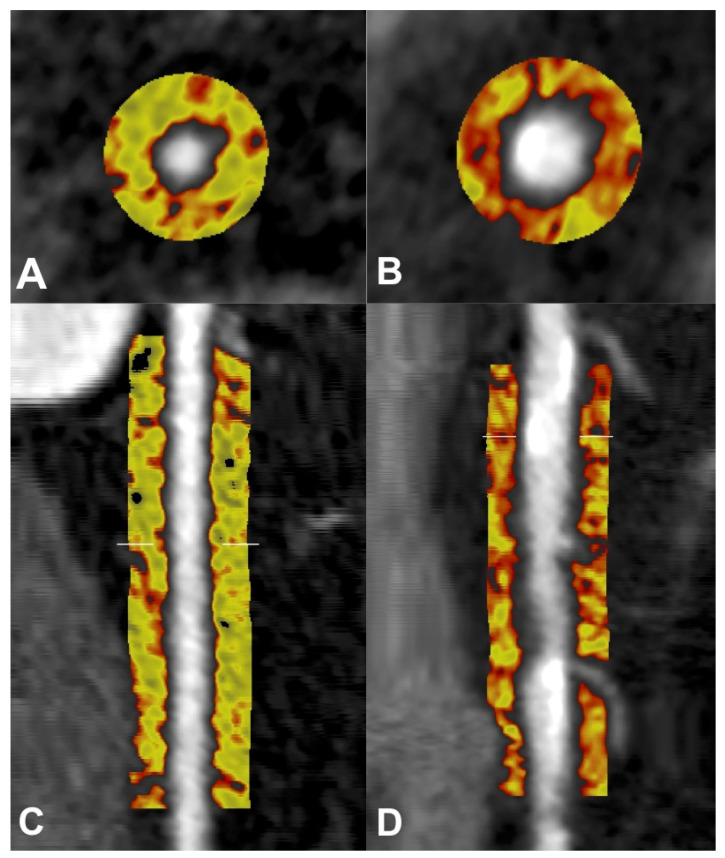
Pericoronary adipose tissue (PCAT) shown in cross-sectional (**A**,**B**) and longitudinal (**C**,**D**) views of the right coronary artery (RCA) on coronary computed tomography angiography (CTA). PCAT in RCA without plaque represented in the left panels (**A**,**C**), and PCAT in RCA with calcified and non-calcified plaque represented in the right panels (**B**,**D**). Colour map describes spectrum of adipose tissue attenuation values in Hounsfield units (HU), ranging from −190 HU (yellow) to −30 HU (red), with higher attenuation values indicating inflammatory changes.

As PVAT depots of the body have a close interaction with the vessels they encase, pericoronary adipose tissue (PCAT) provides the coronary analogue of PVAT and is emerging as a metric of vascular inflammation localised to the coronary tree. The most widely accepted definition of PCAT on coronary CTA is all voxels ranging from −190 to −30 Hounsfield units (HU), within a volume of interest that extends up to an orthogonal distance equivalent to the diameter of the target vessel. PCAT is typically measured around select lesions, or in the proximal segments of the major coronary arteries, particularly the right coronary artery (RCA) due to the low number of side branches, abundance of adipose tissue and uniformity of luminal diameter from its ostial to distal segments [33,55] (Figure 3). These studies were the first to implement the novel pericoronary fat attenuation index (FAI), an AI-driven quantitation of adipose tissue radiodensity that is computationally adjusted for a range of additional factors, such as CT technical parameters and adipocyte morphology [33,55,56,57]. FAI has been shown previously to associate strongly with inflammatory changes to PCAT and the presence of CAD [33]. Moreover, the Cardiovascular RISk Prediction using Computed Tomography (CRISP-CT) study [55] provided further validation by demonstrating the capacity of FAI to predict mortality endpoints in two large prospective cohorts. Mortality endpoints were associated with PCAT measurements in the proximal segments of the major coronary vessels: all-cause and cardiac mortality were associated with PCAT attenuation in the left anterior descending artery (LAD) and RCA, while the same measurement in the left circumflex artery (LCx) was associated with all-cause mortality only. The proximal RCA was selected as a standardised per-patient measurement of PCAT attenuation, representative of inflammation distributed throughout the coronary vasculature, given its association with both study endpoints and the aforementioned anatomical characteristics of the vessel. Importantly, the prognostic value of FAI was lost among those on statin therapy, but retained in those with coronary calcification, demonstrating that coronary CTA attenuation of PCAT reflects vascular inflammatory changes independent of plaque morphology which produces heterogeneity in radiodensity of the wider EAT depot.

These studies have provided strong evidence for a novel form of inflammatory assessment on a per-patient basis in FAI. Since, several observational studies have further underscored the capacity of FAI to dynamically track inflammatory changes within the vasculature, including those induced by therapeutic interventions. Elnabawi et al. [58] performed serial assessment of FAI in the RCA among patients with psoriasis. The authors propensity matched psoriasis patients with and without treatment with anti-inflammatory agents, finding that at one year follow-up, a predictable decline in CRP was accompanied by significantly decreased FAI independent of plaque presence in the treatment group. Furthermore, a recent study by Dai et al. [59] showed that in a statin-naïve population, FAI saw significant reductions after statin treatment within 1.5 years follow-up, particularly among patients with high-risk non-calcified or mixed plaque characteristics. Other high-risk features, however, and calcified plaque did not associate with FAI. Another study by the same group [60] evaluated the relationship between high-risk plaque (HRP), CRP and FAI in a cohort of stable CAD patients. Analysis of FAI was performed around every coronary plaque in each of the three epicardial vessels; it was found that while HRP features were associated with increased CRP, FAI was not able to distinguish between patients with elevated compared to nominal CRP levels. Multivariate predictors of elevated CRP were the presence of low-attenuation plaque (LAP), a high-risk feature detectable on coronary CTA, and percentage of the vessel diameter stenosed, but not FAI. In conjunction, a cohort of patients with myocardial infarction with non-obstructive coronary artery (MINOCA) and Tako-Tsubo Syndrome (TTS) was studied by Gaibazzi et al. [61]. It was found that FAI was higher on both per-patient and per-vessel analyses among MINOCA/TTS patients, and also that LAP was significantly more prevalent among these patients. Finally, in a follow-up study of the CRISP-CT cohort, Oikonomou et al. [62] reported that among large cohorts the adjusted risk of cardiac mortality was highest among patients with high FAI in the RCA and LAD, in subgroups with and without HRP. Moreover, additional analysis adjusted for coronary calcification among patients with no HRP revealed high FAI associated with a higher risk of cardiac mortality than low FAI. Collectively, the current FAI literature delivers a number of key points: (a) anti-inflammatory therapies do indeed effect measurable changes in adipose tissue on coronary CTA, that are also subject to the influence of time; (b) increased systemic inflammation, shown through biomarkers such as CRP, may be poorly related to mechanisms operating specifically within the coronary vasculature; and (c) changes in CT adipose tissue characteristics as a result of coronary inflammation may occur independently to transformations in plaque morphology, including those associated with a greater risk of coronary events.

### 5.3. ‘Crude’ PCAT Attenuation and CAD

Numerous observational studies aside from those listed above have together cultivated a mounting level of evidence into the nature of coronary inflammation shown through PCAT attenuation, albeit without the adjustments afforded by the algorithm that characterises FAI. A range of methodologies have been previously employed to study and classify PCAT on CT, including incremental manual ‘slices’ adipose tissue cross-sectional to the vessel path [63,64,65], ‘fat stranding’ techniques typically evaluated in abdominal CT [66,67], or as a form of quantitative ‘volume’ or ‘thickness’ using axial or reconstructed views [65,68,69]. While these studies are informative, a key limitation is the absence of a clear demarcation between pericoronary and ‘non-pericoronary’ fat, and thus the influence of ‘non-perivascular’ fractions of EAT in analysis cannot be dismissed. Many recent studies, however, have adopted the standardised approach described previously [33,55] and while these studies may assess the ‘unadjusted’ or ‘crude’ form of PCAT attenuation, the employment of a consistent methodology has been conducive to the generation of results that are more readily reproducible and cross-verifiable [70].

PCAT attenuation studies have shown that coronary inflammatory changes may occur incrementally with the burden of CAD and coronary events. Lin et al. [71] found that patients with AMI had distinctly increased PCAT attenuation per-patient in the proximal RCA compared to matched patients with stable CAD and controls without CAD. In adjusted analysis, PCAT attenuation was found to be predicted by stable CAD and MI, and increased PCAT attenuation predicted the presence of AMI, independent of total plaque burden. Additionally, PCAT attenuation did not differ between patients in whom plaque had localised to the RCA, and the authors also found that PCAT attenuation correlated with EAT attenuation only in stable CAD and control patients, highlighting changes specifically within PCAT on coronary CTA occur in highly inflammatory states such as MI, irrespective of the influence of plaque. Conversely, Sugiyama et al. [72] reported that PCAT in the RCA was higher in patients with stable CAD and patients with acute coronary syndrome (ACS) when the culprit lesion was localised to the RCA compared to other vessels; however, PCAT in the RCA was not significantly different between ACS patients and stable CAD patients with an RCA culprit lesion. These seemingly divergent results are comparable, nevertheless, in detailing the specific nature of lesion-specific PCAT attenuation by reflecting localised changes within the coronary vessels whilst also providing further validation of the per-patient PCAT analysis in the RCA as a marker of global coronary inflammation.

Adding to these findings, Goeller et al. [73] demonstrated increased per-patient PCAT attenuation was associated with subsequent increases in NCP and total plaque burden within a mean 3.4 years of follow-up, suggesting potential inflammatory changes associated with the evolution of HRP over time. As discussed, HRP refers to a collection of morphological plaque features that heighten a lesion’s proclivity to rupture and develop into the ‘culprit’ lesions of coronary events such as acute myocardial infarction (AMI) and ACS. These features broadly include plaque phenotypes characterised by low-radiodensity non-calcified, or partially calcified (mixed), composition. HRP features on coronary CTA and their association with ACS have been described in detail previously [74,75], but these inherently describe anatomical changes within the vascular wall that may not reflect early inflammatory mechanisms preceding plaque formation. Accordingly, the relationship between HRP and coronary inflammation has been explored extensively in the recent literature using PCAT attenuation. A small, retrospective study [76] found PCAT attenuation was significantly increased around culprit lesions in patients with ACS, compared to non-culprit lesions within the same patients or highest-grade stenosis lesions in controls with stable CAD. Moreover, within patients with ACS, culprit lesions were associated with greater burden and volume of NCP, which is associated with greater plaque vulnerability. Compared to highest grade stenosis lesions in stable CAD patients, culprit ACS lesions harboured a greater total plaque and NCP burden. Finally, culprit ACS lesions were associated with increased PCAT attenuation per lesion and low-attenuation NCP burden in a multivariable model. These findings are congruent with those of a previously-described study [33], in which a small group of patients with AMI harboured increased PCAT attenuation in stented culprit lesions compared to either stented non-culprit lesions or stented lesions in stable CAD patients. Furthermore, AMI patients with successful 5-week follow-up showed significant reductions in attenuation around the culprit lesion, once again in comparison to stable CAD patients, who had virtually no change in per-lesion attenuation [33]. PCAT attenuation is, therefore, highly dynamic, subject to changes associated with major coronary events in addition to HRP characteristics.

Conversely, in a cohort of stable CAD patients, Kwiecinski et al. [40] found that lesion-specific PCAT attenuation was not significantly different around HRPs compared to non-HRP lesions, but instead increased PCAT attenuation was associated with increased uptake of 18F-NaF on PET-CT. Critically, 18F-NaF activity was only present among 59% of high-risk lesions, and only low-attenuation plaque volume was the only predictor of 18F-NaF uptake in addition to per-lesion PCAT attenuation. This indicates that the unseen inflammatory mechanisms operating within the vasculature are not always captured by the structural changes that comprise classical HRP features. We have recently shown [77] that PCAT attenuation was increased on a per-patient and per-lesion basis in patients with HRP, and that per-patient attenuation was increased in patients with HRP who subsequently develop ACS. Moreover, our time-adjusted analysis revealed ACS was predicted by the presence of HRP as well as PCAT attenuation in the proximal RCA. Thus, where HRP features may provide complementary information to inflammatory status in predicting event risk, PCAT attenuation is useful in its capacity to inform on the extent of coronary inflammation with or without the presence of significant or high-risk lesions.

Additional analyses of these studies indicate that PCAT attenuation may differ between sexes. One study aimed to explore the influence of ethnicity on coronary inflammation, and found that while ethnicity did not contribute to differential PCAT attenuation, male sex did [78]. Likewise, significantly higher PCAT attenuation was found recently in male patients by Sugiyama et al. [72]. Our own study on HRP found that male patients harboured increased PCAT attenuation on both per-patient and per-lesion analyses, and this association persisted in a subgroup of patients with HRP who were then stratified by sex [77]. Interestingly, the magnitude of the difference in crude RCA attenuation in all three of these studies is in the range of 5–6 HU, despite study cohorts being derived from different centres and PCAT attenuation quantified using different software packages. One study provides an exception to this by reporting a 3 HU difference between sexes, although this study evaluated mean attenuation across all three vessels simultaneously as opposed to within the RCA [79]. Serum inflammatory biomarkers are reportedly elevated disproportionately among men [80], but this trend normalises at the onset of menopause in middle-aged women [81,82]. The subject of sex-specific differentials in coronary inflammation shown through PCAT attenuation, adjusted for age and technical parameters, would be a worthy area of investigation and further elucidate the nature of male sex as a traditional risk factor for cardiovascular disease.

### 5.4. PCAT Assessment of Haemodynamically-Significant Lesions

It is important to note that high-risk coronary plaque phenotypes are often non-obstructive in nature [83,84], and thus differential pathophysiological mechanisms may be pertinent to plaque morphology compared to plaque-related stenosis. However, several studies comparing PCAT attenuation with measures of coronary flow and myocardial ischaemia denote the potential role of vascular inflammation in coronary stenosis. Yu et al. [85] found that increased per-lesion PCAT attenuation was associated with lesion-specific ischaemia as shown on a CT-fractional flow reserve (CT-FFR) ≤0.8, but that PCAT attenuation alone was a poor predictor of lesion-specific ischaemia. The predictive value for ischaemic lesions increased significantly in an integrated model incorporating FAI, luminal diameter stenosis and total plaque volume, and was comparable but still marginally inferior to the machine-learning based CT-FFR. Similarly, Hoshino et al. [86] reported increased PCAT attenuation in the proximal LAD was significantly associated with lower FFR, and that increased LAD PCAT attenuation predicted more severely ischaemic lesions shown by FFR ≤ 0.64 and < 0.75, but not FFR ≤0.8. Additionally, a study [87] investigating the relationship between PCAT attenuation and coronary flow reserve (CFR) on PET imaging found that PCAT attenuation was a significant predictor of decreased CFR after adjustment for risk factors and plaque morphology. Moreover, subgroup analyses revealed CFR was significantly lower in patients with increased PCAT attenuation without obstructive CAD, or with coronary calcium score (CCS) <100. It is clear that the interaction between coronary flow, plaque characteristics, and PCAT attenuation is highly complex, but these studies provide a number of consistent results. Coronary inflammation may indeed be observable among patients with impaired coronary flow, mechanistically explained by the disruption of PCAT’s role in modulating vascular tone due to focal inflammation. However, the observational nature of these studies means the directionality of this relationship—whether PCAT attenuation is the cause or effect of lesion-induced ischaemia—yet remains unclear.

### 5.5. PCAT Assessment in Non-Atherosclerotic Disease States

Therefore, both adjusted and ‘crude’ PCAT assessment have demonstrated distinctly increased coronary inflammation in patients with developing high-risk lesions or major coronary events, collectively providing further validation for the potential clinical utility of this technique. In addition to these studies on orthodox coronary atherosclerosis, PCAT attenuation has been explored in a number other coronary and inflammatory disease states, including vasospastic angina, atherosclerotic intraplaque cholesterol crystals, and spontaneous coronary artery dissection. Firstly, vasospastic angina (VSA) has received increasing attention in several independent observational studies. Ueno et al. [88] found that in a cohort of 88 patients with and without VSA, patients with VSA were found to have increased PCAT attenuation, and that attenuation of PCAT in the RCA was superior as a predictor of VSA than in the other two epicardial vessels. Likewise, a case study [89] in a woman with MINOCA possibly induced by VSA found a significant decrease in PCAT attenuation in the LAD on two-year follow-up, although the presence of acute inflammatory changes associated with AMI itself may have contributed to the increased attenuation at initial presentation. Nevertheless, the plausibility of inflammatory PCAT changes in this condition is evidenced by demonstrably increased 18F-FDG uptake in VSA patients which is then mitigated by medical therapy [90].

Secondly, cholesterol crystals refers to high intensity regions within a lipid-rich plaque visualised on optical coherence tomography (OCT) that triggers inflammatory activity and plaque instability [91]. A small study featured assessment of PCAT around coronary plaques, as well as in coronary segments harbouring plaque, and finally on a per-patient analysis in the proximal RCA among a cohort of patients with intraplaque cholesterol crystals. All three metrics of PCAT were increased relative to the presence of lesions with cholesterol crystals; increased attenuation was found around lesions and in coronary segments with lesions in which cholesterol crystals were detected, while patients with cholesterol crystals had higher PCAT attenuation than those who did not. Moreover, per-lesion PCAT attenuation increased proportional to the number of intraplaque cholesterol crystals detected [92].

Lastly, spontaneous coronary artery dissection (SCAD) is a non-atherosclerotic condition of unknown aetiology typified by formation of an intramural haematoma, typically within the outermost portion of the tunica media, in turn reducing luminal diameter and flow [93]. In a single-centre retrospective study of a small group of patients with SCAD, we reported no significant difference in PCAT attenuation between SCAD cases and asymptomatic controls [94], implying a potentially limited role played by coronary inflammation in this condition, although the size of our cohort due to the rarity of SCAD necessitates further investigation in larger cross-sectional studies.

### 5.6. Technical Parameter Influence in PCAT Assessment

As an emerging non-invasive tool, the utility of PCAT requires validation not only across different inflammatory conditions, but also across multiple scanners and technical parameters involved in CT imaging. Given the nature of coronary CTA as one of the first-line imaging modalities with wide usage in assessment of suspected CAD, PCAT attenuation studies have thus far analysed primarily contrast-enhanced CT acquisitions in both retrospective and prospective cohorts. Assessing the impact of factors extraneous to disease pathophysiology on PCAT attenuation has formed the basis of a number of recent studies, which have explicated the role played by the use of contrast on PCAT attenuation. Almeida et al. [95] studied patients with CAD who underwent pre- and post-contrast scans, which were used as points of comparison for attenuation and volume differences owed to the impact of contrast enhancement. Pre-contrast scans had significantly lower PCAT attenuation of approximately 3–4 HU, as well as lower PCAT volume, compared to post-contrast scans. Nevertheless, there was a high degree of correlation between pre- and post-contrast PCAT attenuation and volume measurements, with excellent inter- and intra-observer reliability. Additionally, the authors found a high correlation between two software packages (TeraRecon and AutoPlaque) in PCAT quantification. Among contrast-enhanced scans, Ma et al. [79] evaluated the impact of tube voltage on PCAT attenuation. In this study, PCAT attenuation was averaged across 10mm segments derived from each major epicardial vessel, up to 1mm orthogonally from the vessel wall as opposed to the 2–3 mm used widely in the literature [33,55] as the LAD and LCx are encased in comparably less adipose tissue than the RCA. It was found in this study that mean PCAT attenuation was predicted by tube voltage in conjunction with age and sex, and that mean PCAT attenuation as well as EAT volume progressively increased with tube voltage, which ranged from 70 to 120 kV.

Additional evidence for the influence of contrast-enhancement arises from the findings of studies utilising alternative forms of PCAT quantification on non-contrast CT [64,69]. In a group of patients without CAD, Hell et al. [64] found that PCAT attenuation, shown through cross-sections of the coronary vessels per patient, significantly decreased from the proximal to mid LAD, while no such difference was found around the segments in the RCA or LCx. Balcer et al. [69] defined adipose tissue as voxels between −195 to −45 HU within a manually-drawn region of interest of each vessel on axial non-contrast CT, but still found significantly decreased attenuation between the proximal and mid LAD. Anatomically, luminal diameter decreases from ostium to periphery in the LAD and LCx, while the RCA remains consistent in its diameter until its bifurcation. This may account for the increased attenuation in the proximal LAD found in these studies, but it is unclear why significant decreases in PCAT attenuation around the LCx were not found. Collectively, these findings underscore not only the importance of adhering to standardised PCAT quantification, but the necessity of maintaining consistent scan parameters across patient cohorts in which PCAT may be evaluated.

### 5.7. Current Limitations and Future Directions

Current knowledge on PCAT attenuation as an imaging biomarker of inflammation is, therefore, ever-expanding, but is not without some limitations. Differences in PCAT attenuation owed to epicardial and overall adiposity [64], as well as due to sex [72,77,78,79], call for future studies to adjust for these potential patient-specific confounders. While the impact of specific scan parameters on PCAT attenuation has been increasingly evaluated, validation of ‘crude’ PCAT assessment across different scanners has yet to be explored. As discussed, PCAT attenuation has been assessed previously using a range of methodologies [63,64,65,66,67,68,69], and this has been to a large extent accounted for via the use of validated per-patient and per-lesion forms of assessment. However, there is some heterogeneity as to the degree to which plaque within the RCA may affect the per-patient assessment of PCAT in this vessel [71,72]. Moreover, the present definition of PCAT (adipose tissue within a distance equivalent to the vessel diameter) includes an inherently larger volume of adipose tissue in the RCA than in its counterpart vessels. The consistent luminal diameter of the RCA ensures that adipocytes within up to approximately 2–3 mm from the vessel wall are considered PCAT throughout the vessel’s passage; conversely, the LAD and LCx are encased in less adipose tissue overall, and decreases in luminal diameter mean a progressively decreasing volume of adipose tissue volume in the mid-to-distal segments of these vessels falls within the classification of PCAT. In theory, the broader inclusion of adipose tissue around the RCA indicates PCAT assessment in this vessel may include adipocytes that are distinctly less ‘inflamed’, while PCAT in the LAD or LCx would consist only of adipocytes that are most proximal to the vessel wall. In this regard, alternative methodologies consistent with the theoretical framework outlined previously [33,55] would be worthy of consideration. For example, a recent study [79] evaluated per-patient PCAT attenuation as the mean attenuation of adipose tissue within a region of 1 mm thickness around all three coronary vessels, and thus a consistent volume of PCAT being assessed for each vessel was ensured. Nevertheless, extensive validation and a wealth of literature highlights the utility of the current methods of PCAT assessment at the per-patient and per-lesion level [33,55].

## 6. Conclusions

The recent years have witnessed an upsurge in interest towards coronary inflammation and its role in early and advanced stages of atherosclerosis. The current literature provides a greater understanding of the role played by insidious inflammatory mechanisms within the coronary vasculature, and emphasises the importance of assessing unseen biological processes preceding and precipitating the development of significant coronary plaques. PCAT attenuation on coronary CTA therein has presented a non-invasive and widely-accessible surrogate marker of coronary inflammation, capable of mapping inflammatory changes that are associated with CAD in both stable and ‘vulnerable’ populations. The current array of publications that have investigated PCAT attenuation highlight its feasibility in CAD assessment, as well as in tracking inflammation in response to medical therapy and coronary conditions with a limited involvement of CAD. While standardised CT thresholds of inflammation are yet to be established, the potential for risk stratification based on non-invasive PCAT assessment presents a powerful avenue through which primary prevention initiatives may be enhanced via the detection of coronary inflammation early in the pathogenesis of atherosclerosis.

## Data Availability

Not applicable.
